# Infrared thermography detects febrile and behavioural responses to vaccination of weaned piglets

**DOI:** 10.1017/S1751731114002481

**Published:** 2014-10-02

**Authors:** N. J. Cook, B. Chabot, T. Lui, C. J. Bench, A. L. Schaefer

**Affiliations:** 1 Alberta Agriculture and Rural Development, Livestock Research and Extension Division, Lacombe Research Centre, 6000 C&E Trail, Lacombe, Alberta, Canada T4L 1W1; 2 Agriculture and Agri-Food Canada, Lacombe Research Centre, 6000 C&E Trail, Lacombe, Alberta, Canada T4L 1W1; 3 University of Alberta, 3-10G Agriculture/Forestry Centre, University of Alberta, Edmonton, Alberta, Canada T6G 2P5

**Keywords:** pigs, vaccination, febrile response, infrared thermography

## Abstract

An automated, non-invasive system for monitoring of thermoregulation has the potential to mitigate swine diseases through earlier detection. Measurement of radiated temperature of groups of animals by infrared thermography (IRT) is an essential component of such a system. This study reports on the feasibility of monitoring the radiated temperature of groups of animals as a biomarker of immune response using vaccination as a model for febrile disease. In Study A, weaned pigs were either treated with an intramuscular vaccine (FarrowSure Gold), a sham injection of 0.9% saline or left as untreated controls. An infrared thermal camera (FLIR A320) was fixed to the ceiling directly above the pen of animals, and recorded infrared images of the treatment groups at 5 min intervals. The effect on temperature of the spatial distribution of pigs within the pen was significant, with higher temperatures recorded when pigs were grouped together into a single cluster. A higher frequency of clustering behaviour was observed in vaccinated animals compared with controls during a period of the afternoon ~4 to 7 h post-vaccination. The daily mean of the maximum image temperature was significantly higher in vaccinated animals compared with control and sham-treated animals. In the vaccination treated group, the 24 h mean of the maximum temperature was significantly higher during the post-vaccination period compared with the 24 h period before vaccination. Increased temperature in the vaccinated animals occurred from ~3 h, peaked at ~10 h, and remained elevated for up to 20 h post-vaccination. In Study B, the effect of prevalence was tested in terms of the difference in maximum temperature between control and vaccination days. A thermal response to vaccination was detected in a pen of 24 to 26 animals when <10% of the animals were vaccinated. The results support the concept of radiated temperature measurements of groups of animals by IRT as a screening tool for febrile diseases in pig barns.

## Implications

Measurements of radiated temperature of a group of pigs using infrared thermal cameras can detect febrile responses to vaccination, even when the prevalence of vaccinated animals in the group is <10%. Thermal images can be used to quantify clustering behaviour and this behaviour is associated with a febrile condition. Thus, it may be possible to combine temperature and behavioural measurements from thermal images into an automated disease detection system in pig barns.

## Introduction

Infrared thermography (IRT) is a non-contact method of measuring the radiated surface temperature of animals. This gives rise to the concept of IRT as a remote method of disease surveillance, eliminating the need to restrain animals in order to measure their temperature. The technology has mostly been used to screen people at airports for the purposes of disease surveillance (Bitar, 2009), but also has a role in veterinary medical diagnostics (McCafferty, 2007). From the veterinary perspective, IRT has mostly been used to identify localized areas of infection or inflammation, such as mastitis (Colak *et al.*, 2008; Hovinen *et al.*, 2008), foot and mouth disease (Rainwater-Lovett *et al.*, 2009), and hot *v.* cold branding in cattle (Schwartzkopf-Genswein and Stookey, 1997). IRT has also been applied to the detection of febrile responses to systemic illness of cattle, including bovine viral diarrhoea (Schaefer *et al.*, 2004) and respiratory disorders (Schaefer *et al.*, 2007). In pigs inoculated with *Actinobacillus pleuropneumoniae*, mean body surface temperatures were indicative of infection (Loughmiller *et al.*, 2001). An automated system for measurements on individual animals, utilizing radiofrequency identification (RFID) tags for identification and for triggering the recording of thermal images, has been demonstrated in feedlot cattle (Schaefer *et al.*, 2012). However, the system relies on animals visiting a facility such as a water or feed station, which introduces a source of confounding variation since such visits may be relatively infrequent, or may occur at different times of the day. In the above examples, measurements of radiated temperatures were made on individual animals. The present proof-of-concept study tests a much simpler system of taking thermal images of groups of animals on a regular and frequent basis for the purpose of detecting febrile responses.

The group temperature of pens of pigs, measured using a hand-held infrared camera, was reported to be predictive of illness 2 days before mortality within the group (Friendship *et al.*, 2009). However, taking thermal images with a hand-held camera compromises the precision and accuracy of the measurements, and is impractical from a disease surveillance perspective because it requires the camera operator to visit the same pens at least daily, and much more frequently if the efficacy of the measurement is to be optimized. For these reasons, an automated system that takes frequent measurements is a prerequisite for disease detection and surveillance. However, there are several problems that need to be overcome for such a system to have practical application. Images of groups of animals may result in a loss in test sensitivity because changes in the temperature of an individual may be masked by the temperature of the group. Also, much of the image content is associated with the surrounding environment rather than the animals, for example floor, walls, etc. Consequently, images of a group will contain temperature information that is irrelevant, and the temperature data acquired from the image must take these factors into account.

The objectives of the current studies were first to test if changes in temperature in response to immune challenge could be detected by thermal images of groups of animals. Second, to address the question of prevalence, that is how many animals within a group would need to exhibit thermal responses before those responses were detectable? Vaccination was used to stimulate immune reactions, and prevalence was modelled by varying the numbers of pigs in the group that received the vaccine.

## Material and methods

Pigs were cared for in accordance with the Canadian Council on Animal Care in Science (2009), and the study was approved by the Animal Welfare Committee of the Lacombe Research Centre.

### Study A: clustering behaviour, vaccination and radiated temperature

Newly weaned (Large White Landrace×Duroc) piglets (*n*=210), aged 21 to 28 days, with a mean (s.e.) weight=8.91 (0.22) kg were housed in groups of seven in a pen measuring 6×4 feet (24 sq ft) with fully slatted plastic flooring. Pigs were given *ad libitum* access to water, a post-weaning, complete, crumble feed and an electrolyte solution (Vetoquinol Lavaltrie, QC, Canada) containing vitamins and electrolytes for correcting dehydration and electrolyte imbalances during periods of stress such as weaning. Pigs were allowed to acclimatize to the test pen for a minimum of 3 days before the onset of image collection and up to 5 days before vaccination. There were 7 pigs in each treatment group and a total to 10 replications (Reps) of the experiment. Thus, there were 70 pigs used per treatment group. The vaccination treatment group (Vac, *n*=70) received an intramuscular injection of the vaccine FarrowSure Gold (Zoetis Inc., Kalamazoo, MI, USA). This is a three-way vaccination for porcine parvovirus, erysipelas caused by *Erysipelothrix rhusiopathiae*, and leptospirosis caused by *Leptospira bratislava*, *Leptospira canicola*, *Leptospira grippotyphosa*, *Leptospira hardjo*, *Leptospira icterohaemorrhagiae* and *Leptospira pomona.* The sham treatment group (Sham, *n*=70) received an intramuscular injection (2 ml) of 0.9% saline, and a control group (Con, *n*=70) did not receive any treatment. Infrared images were recorded at 5 min intervals for 2 days, representing a control day and test day in the Vac and Sham groups, and 2 control days in the Con group. Images recorded before administration of vaccination or sham treatments were designated as Vac Con and Sham Con, respectively. Images recorded over the 24 h period following treatments were designated Vac Trt and Sham Trt. This allowed for comparisons of responses within and across treatment groups. Treatments were conducted in a revolving order over 10 Reps within the same pen, over a period totalling 18 months.

**Figure 1:**
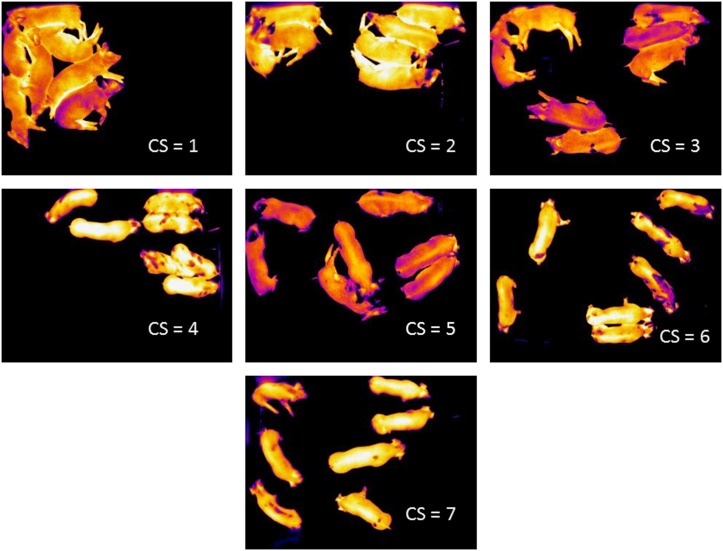
Examples of the spatial distributions of pigs within the pen showing cluster score categories 1 to 7. Cluster scores of 1, 2 and 3 were combined to give a high cluster score (HCS) category, and cluster scores of 4, 5, 6 and 7 were combined to give a low cluster score (LCS) category.

Thermographic images were taken using a single radiometric infrared camera (FLIR A320; FLIR Vision Systems, Burlington, ON, Canada). The camera was mounted on the ceiling of the nursery barn directly overhead of the test pen, and 9 ft. from the camera lens to the floor of the pen. Infrared images were analysed for temperature variables using ThermoCAM Research Pro 2.7 (FLIR Vision Systems). This program allowed for temperature measurements of the whole image or parts of the image that were delineated using shapes or free-drawing tools. The temperature data obtained from the images were the minimum, maximum, range and mean. Infrared images included parts of the pen such as the floor, walls, feeder and electrolyte solution, as well as less consistent variables such as the heat imprint of the pigs on the floor, heat reflections of pigs on the solid, plastic walls, recent depositions of urine or faeces, and occasionally the presence of mice in the pen. Consequently, the minimum, mean and range of image temperatures were influenced by these extraneous factors. Attempts were made to eliminate these variables by setting a threshold temperature that removed the background and permitted measurement of the mean pig temperature. Figure 1 shows images in which a threshold temperature was set to remove the background. Unfortunately, the mean pig temperature became a function of the method used to determine the threshold value and as a consequence only the maximum image temperature was independent of these confounding variables. Note that the maximum image temperature was always obtained from the pigs since these were the warmest objects in the pen, and was therefore used for statistical and comparative purposes.

The spatial distribution of pigs within the image could have affected the temperature measurements. Therefore, the distributions of pigs were assessed using a cluster score (CS). Examples of the CS of the seven piglets are shown in Figure 1. If all pigs were touching and huddled into one area of the image then CS=1, and if all pigs were individually distributed throughout the image then CS=7. Thus, individual pigs could be a ‘cluster’. However, if two pigs were touching but standing and aligned end-to-end they were not considered to be clustering unless a minimum of half of their body lengths were in contact. It was recognized that the distribution of pigs within the same CS category could be different. Thus, there was only one possible distribution of pigs if CS=1, 6 or 7, but for CS=2, 3, 4 and 5, pigs could have two or three different distributions within each of these scores. For example, CS=2 can have possible distributions of 6+1, 5+2 and 4+3. Cluster scoring was a relatively simple method of representing spatial distribution but served the purpose of testing if spatial distribution affected the measured temperature. High and low clustering activities were defined by combining CS1, CS2 and CS3 into a high cluster score group (HCS), and CS4, CS5, CS6 and CS7 into a low cluster score group (LCS).

Image time was expressed as clock time and treatment time. The actual clock time for all images was recorded automatically by the infrared camera. To allow for the compilation of time-matched data across Reps, clock times were rounded to the nearest 5 min, for example 9:32:29 rounded to 0930 h, and 9:32:30 rounded to 0935 h. Treatment time was expressed in minutes relative to the administration of either the vaccine or saline injection at Time 0. In the Con treatment, Time 0 was taken as the image recorded closest to the same clock time as the Time 0 image in the Vac treatment for that Rep. Temperature responses to treatments were illustrated over 24 h periods, and the mean of the maximum temperature over these 24 h periods was used to test for treatment effects.

Environmental effects on the measurement of animal temperature were assessed by comparing the maximum animal temperatures with air temperature (°C), humidity (%) and atmospheric pressure (in Hg) recorded with a Kestral 4000 weather meter (Optimum Energy Products, Calgary, AB, Canada) that was suspended above the pen but out of frame of the infrared camera. There were a total of 3771 paired observations between environmental measurements and pig temperature on the same control and treatment days recorded over 9 Reps.

### Study B: estimating the effect of prevalence on the measurement of group temperature

Prevalence was defined as the percentage of animals in a pen that had been vaccinated. Groups (*n*=16) of weaned piglets (*n*=23 to 28 per group) with a mean (s.e.) weight of 8.01 (0.13) kg were housed in a pen of dimensions 6×14 ft (84 sq ft) with fully slatted plastic flooring and plastic walls. The animals were separate groups to those used in Study A. The access to feed, water and electrolyte solution, and the vaccination protocol, were the same as was stated for Study A. Owing to the larger pen area, the infrared camera (FLIR A320) was fitted with a 90° lens. Images recorded with this lens exhibited edge distortion such that it was not possible to distinguish clustering behaviour, and consequently CSs were not recorded for this part of the study.

Pigs were allowed to acclimatize to the experimental pen for 3 days. Thereafter, images were recorded at 5 min intervals over 3 consecutive days with vaccination on the second day. In each of the groups a different number of pigs were vaccinated with FarrowSure Gold, providing a range of prevalence of vaccinated animals of 7.1% to 70.8%. Groups with prevalence of 33.33% (9 out 27 pigs), 41.67% (10 out of 24 pigs), 47.82% (11 out of 23 pigs) and 66.66% (18 out of 27) were repeated twice.

### Statistical analyses

Statistical analyses were performed using JMP v10 (SAS, Cary, NC, USA). In Study A, one-way ANOVA tested for differences in the mean of the maximum image temperatures between CSs. Mixed model analysis was used to test the effects of treatment time, and image type (Vac Con, Vac Trt, Sham Con, Sham Trt and Con) and their interactions (time×type), with Rep as a random effect in the model. The response phases were taken as the 24 h period following treatment (Vac Trt and Sham Trt) and the control phases as the 24 h period before treatments (Vac Con and Sham Con). A comparable 24 h control-phase was determined for the Con animals. Contingency table and *χ* ^2^ analyses tested for differences in the distribution of CS and frequency of clustering behaviour between treatments.

In Study B, the response parameter was taken as the difference in the maximum temperature (Temp Diff) between post-vaccination and pre-vaccination conditions for time-matched images over consecutive 24 h periods. The mean Temp Diff over the 24 h period was taken as the response variable. ANOVA was used to test for the effect of prevalence on the Temp Diff variable, and a bivariate fit of prevalence *v*. mean Temp Diff illustrated this relationship.

## Results and discussion

### Environmental variables *v.* maximum pig temperature

There were 3771 matched observations for maximum pig temperature and the environmental variables; air temperature, humidity and atmospheric pressure. Table 1 gives the mean (s.e.) for each of the variables and the bivariate fit analyses (*R* ^2^ values) for maximum pig temperature *v*. environmental variables. Further analysis of the data revealed air temperature followed a pattern of being lower at night and higher during the day. The lowest hourly mean (s.e.) air temperature was 25.56 (0.12)°C between 0400 and 0459 h, and the highest was 26.6 (0.1)°C between 1400 and 1459 h. The mean (s.e.) difference in air temperature between these times was 1.04 (0.16)°C (*P<*0.0001). The maximum pig temperature exhibited the opposite pattern. The highest hourly mean (s.e.) pig temperature was 38.37 (0.06)°C recorded between 0100 and 0159 h, and the lowest was 37.64 (0.06)°C between 1500 and 1559 h. The mean (s.e.) difference was 0.72 (0.08)°C (*P<*0.0001). Thus, over a 24 h period these variables were changing in opposite directions relative to one another, explaining why there was a weak negative association between maximum pig temperature and air temperature. These observations confirmed that in the present study, where the environmental temperature of the nursery room was controlled within narrow limits, it was not necessary to adjust the measurement of pig temperature to account for variations in the environmental conditions. However, it is important to recognize that this finding is limited to the specific environmental conditions of the present study in which air temperature was maintained with narrow limits. A significant, positive linear regression and correlation between skin surface temperature, as measured by IRT, and air temperature has been observed for ranges in air temperature of 15 to 25°C (Geers *et al*., 1987), and 10 to 32°C (Loughmiller *et al.*, 2001).

**Table 1:** The mean (s.e.) of the maximum pig temperature, air temperature, humidity, heat index and air pressure in paired observations (*n*=3771) across nine replications of Study A

Pig and environmental variables	Mean	s.e.	Bivariate fit (*R* ^2^)^1^
Maximum pig temperature (°C)^2^	38.02	0.01	
Air temperature (°C)	25.89	0.03	−0.0775^a^
Relative humidity (%)	33.72	0.14	0.0331
Atmospheric pressure (in Hg)	26.90	0.01	−0.0071

^a^Bivariate fit of maximum pig temperature with environmental variables that were statistically significant at *P*<0.05.

1 Bivariate fits of the maximum pig temperatures with the environmental variables (air temperature, relative humidity and atmospheric pressure).

2 Maximum recorded temperature in the thermal image. Note that this was always a pig.

### Study A: the effects of clustering behaviour

Figure 1 shows examples of the clustering behaviour of pigs and the CS categories. Two questions were addressed with regard to clustering behaviour. First, did clustering affect temperature measurements, and second did treatments affect clustering behaviours? Table 2 gives the number of images, and the mean (s.e.) of the maximum temperature for CS within treatment groups. The total number of CS observations was 13 202 of which 34.7% were CS1, declining in successive CS categories to 2.7% for CS7. CSs of CS1, CS2 and CS3 (HCS) were associated with inactivity, and LCS was indicative of activity (Figure 1). The HCS=71.5% of all observations, indicating that most of the time pigs were exhibiting huddling behaviour (usually sleeping). One-way ANOVA indicated that CS had a significant effect on maximum image temperatures across a combination of all treatment groups (*P*<0.0001), and within individual treatment groups (*P*<0.0001) (Table 2). Across all treatment groups and within individual treatments there were significant declines in the maximum image temperatures with increasing CS. Thus, as the spatial distribution of pigs increased there was a decline in the maximum temperature.

**Table 2:** The numbers of observations and the means (s.e.) of the maximum pig temperatures within cluster score categories and treatment groups in Study A

	Treatment groups											
	All groups			Con			Sham			Vac		
CS	*n*	Mean	s.e.	*n*	Mean	s.e.	*n*	Mean	s.e.	*n*	Mean	s.e.
1	4577	38.57^a^	0.02	1271	38.32^a^	0.04	1652	38.46^a^	0.02	1646	38.88^a^	0.02
2	2823	38.32^b^	0.02	731	38.21^a,b^	0.05	928	38.30^b^	0.03	1133	38.39^b^	0.03
3	2045	38.18^c^	0.02	592	37.07^b,c^	0.05	650	38.09^c^	0.03	772	38.21^c^	0.03
4	1478	38.0^d^	0.03	415	37.94^c,d^	0.06	495	37.96^d^	0.04	526	38.09^d^	0.04
5	1153	37.78^e^	0.03	388	37.84^d,e^	0.06	406	37.68^e^	0.04	347	37.84^e^	0.04
6	766	37.66^f^	0.04	259	37.73^e^	0.08	252	37.58^e,f^	0.05	247	37.68^f^	0.05
7	360	37.47^g^	0.05	127	37.61^e^	0.11	145	37.42^f^	0.07	87	37.37^g^	0.08

^a,b,c,d,e,f,g^Mean temperature measurements within a column not sharing the same superscripts differ significantly at *P*<0.05.

**Figure 2:**
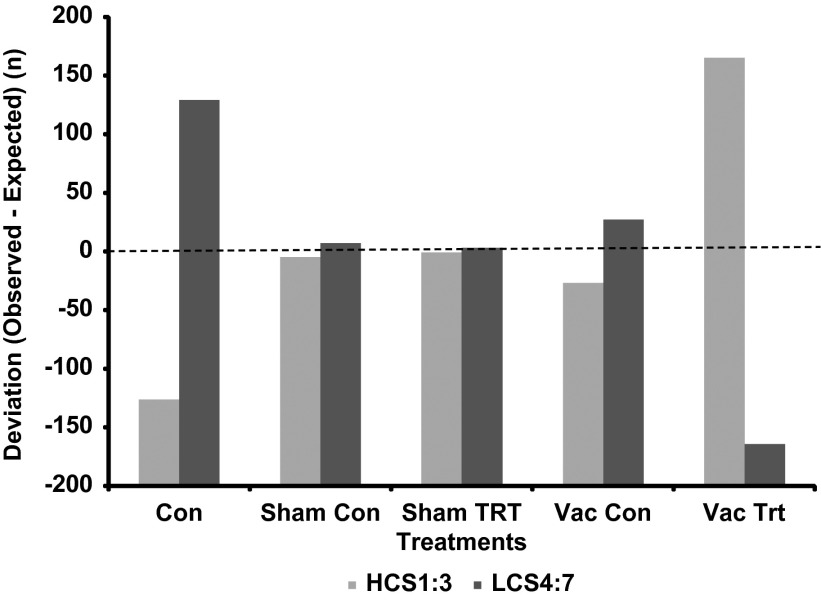
The numbers of deviations in high cluster score (HCS) and low cluster score (LCS) categories for treatment groups in Study A. Positive values show a greater number of observations in that cluster score (CS) category than would be expected from *χ* ^2^ distributions. Negative values show lower than expected numbers of observations.

The effect of treatment on CS was tested by comparing the differences in the numbers of observations for CS categories between treatment groups using a contingency table and *χ* ^2^ analysis. This indicated that the numbers of observations in each CS category differed significantly between treatment groups (*χ* ^2^=183.4, *P*<0.0001). Contingency table analysis gave the predicted and observed number of observations for each CS category within treatment groups. The difference between the predicted and observed number of observation was termed the ‘deviation’. In the Vac Trt group, deviations for the CS1, CS2 and CS3 categories were more than would be expected. Note that these categories were the ones that made up the HCS grouping. To illustrate the effect of treatment on clustering behaviour, it was necessary to correct for the imbalance in the total numbers of images recorded in different groups. Thus, the number of observations in each CS was expressed as a percentage of the total number of observations for each treatment group. Data were further consolidated by combining CS into HCS and LCS as previously described. Figure 2 shows the deviations in observations for HCS and LCS in each treatment group. Note that the distributions of the deviations for the HCS and LCS were opposite in the Vac Trt group relative to all other treatment groups, and that there were more HCS and less LCS in the Vac Trt group than would be expected.

**Figure 3:**
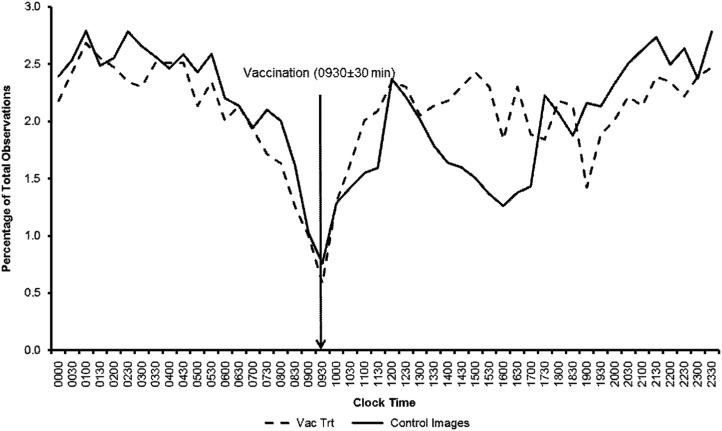
The percentages of high cluster scores (HCS) over 30 min intervals in vaccinated animals and a combination of all control images (Con, Vac Con and Sham Con) in Study A.

Figure 3 illustrates the changes in the percentage distribution of HCS relative to the time of vaccination, and compares the Vac Trt images with the mean for all the Con images, that is Vac Con, Sham Con and Con. These data were plotted by clock time to illustrate the time of day effect on clustering behaviour. The percentage of HCS was high during the night and started to decline from ~0500 h reaching a trough in HCS at the time of vaccination (0930 h). It was coincidental that the trough in HCS in the Con animals matched the time of vaccination since these animals were left undisturbed, but was probably due to the activity of staff entering the nursery room to vaccinate other animals, and fill feeders. Following vaccination there was a sharp increase in HCS that reached a peak at ~1200 h. Thereafter, the Con animals exhibited a second trough in the afternoon between 1400 and 1700 h, whereas in the Vac animals HCS remained relatively high during this period. Paired *t*-tests showed that percentage of HCS in the Vac treatment (31.7%) was significantly higher (*P<*0.0002) than the Con treatments (25.2%) during the period 0930 to 1700 h, that is up to 7.5 h post-vaccination.

Cluster analysis revealed that the spatial distribution of pigs in group images had an effect on the measured temperature. Furthermore, that vaccination had an effect on clustering behaviour such that vaccinated pigs clustered together more, particularly from the time of vaccination at 0930 h up to 1700 h, and this behaviour coincided with the peak in the maximum temperature. We can only speculate on the reasons why vaccination should induce more clustering behaviour. It may be that the vaccinated animals felt sick, or stressed, and derived comfort from huddling together. Behavioural change in response to disease is well recognized as an adaptive mechanism. For example, pigs experimentally inoculated with *Mycoplasma hyopnuemoniae* and porcine reproductive and respiratory syndrome virus exhibited reduction in activity and an increase in huddling behaviour (Escobar *et al.*, 2007).

### Study A: vaccination effects on radiated temperature

**Figure 4:**
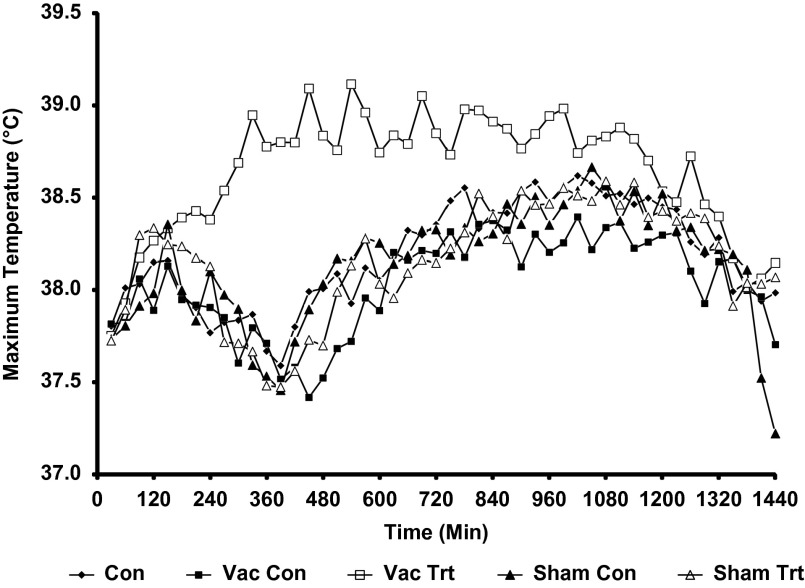
The mean of the maximum pig temperature for 30 min intervals relative to vaccination and sham injections (Time 0) in all treatment groups in Study A. The control group of animals are designated as Con, and the Vac Con and Sham Con were images collected on control days for the Sham and Vac treatment groups. Sham Trt and Vac Trt were images collected on Sham and Vac treatment days.

The thermal response to vaccination is illustrated in Figure 4. The temperature data are presented as the mean for 30 min sampling periods, that is six images per 30 min period. Mixed model analysis revealed that time, treatment and their interaction (time×treatment) were significant effects for the maximum image temperature (*P*<0.0001). Least square 24 h mean temperature in the Vac Trt images (38.64°C) was significantly higher (*P*<0.0001) than Vac Con (38.05°C), and significantly higher (*P*<0.05) than all other treatment groups; Con=38.18°C, Sham Con=38.19°C, Sham Trt=38.18°C. There were no differences in the maximum temperature between the Con, Sham Con and Sham Trt images. However, the Vac Con images exhibited a lower maximum temperature than all other groups (*P*<0.05).

Study A demonstrated that vaccination caused a significant thermal response in pigs, which was evident within 3 h, peaked at ~10 h, and lasted for up to 20 h post-vaccination. Using measurements of radiated temperature by IRT and rectal temperature on individual animals, Loughmiller *et al.*, 2001 describe a similar time course for the febrile response (6 to 18 h) following acute challenge with *A. pleuropneumoniae*. The present study provides proof of concept that measurement of radiated temperature of groups of animals rather than individuals may provide a detection system for febrile responses.

Vaccination affected clustering behaviour such that animals that received the vaccine exhibited more clustering compared to control conditions, particularly up to 7.5 h post-vaccination. Thus, a combination of thermal measurements and clustering behaviour, derived from the same thermal images, may optimize the diagnostic capabilities of IRT for febrile diseases. At the least, the determination of clustering in thermal images could be used to select those images most appropriate for thermal analysis. For example, limiting thermal analyses to those images in the CS1 category would reduce variation in the temperature measurement due to spatial distribution. If the prevalence of the CS1 category was reduced due to less huddling behaviour at higher environmental temperatures, then thermal analysis could be limited to those images in which the CS category exhibited the least variation, or the category that was most prevalent.

### Study B: the effect of prevalence

**Figure 5:**
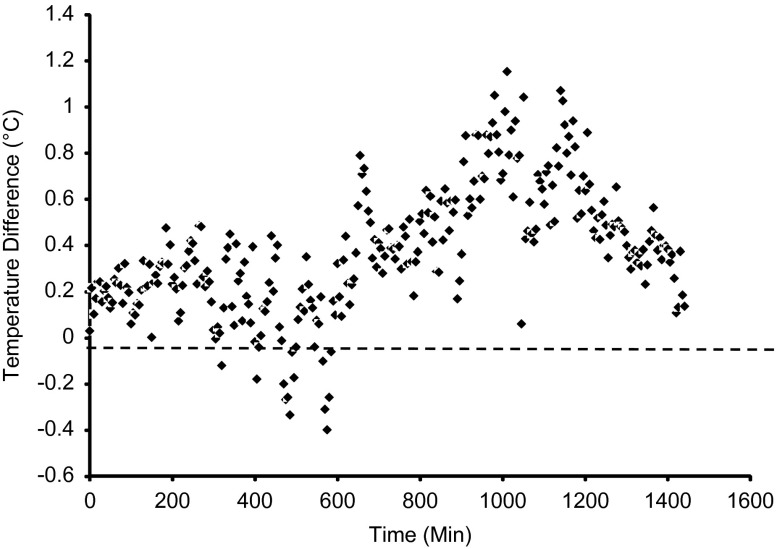
The mean of the Temp Diff parameter for pig maximum temperature at each 5 min sampling interval for all levels of prevalence in Study B. A positive value meant that the temperature in the post-vaccination image was higher than in the pre-vaccination image.

**Figure 6:**
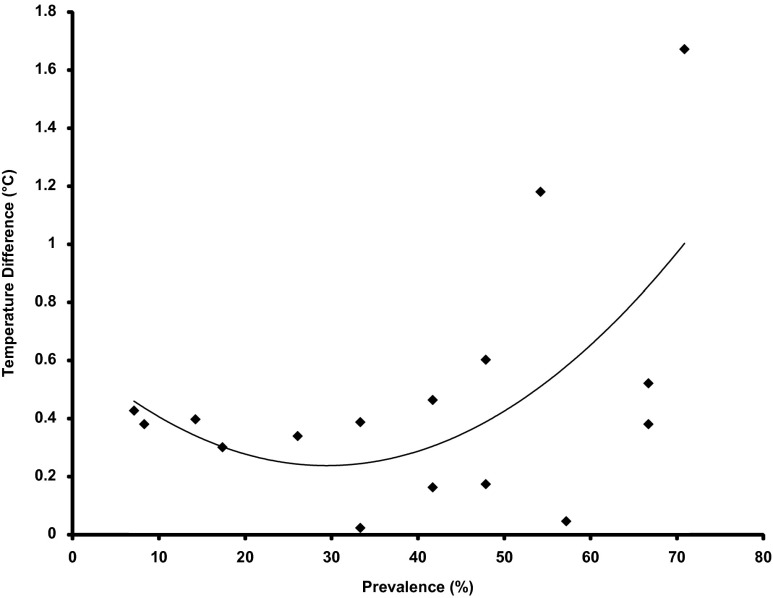
The relationship between prevalence and the Temp Diff parameter for pig maximum temperature in Study B.

Figure 5 shows the mean Temp Diff across all levels of prevalence. Note that the mean level of prevalence across Reps was 39.9%, representing 157 out of 393 pigs in total. One-way ANOVA revealed a trend in the variances across the different levels of prevalence (*P*<0.08). Similarly, the bivariate fit of prevalence and 24 h mean Temp Diff (Figure 6) indicated that the relationship could be described by a second order polynomial curve fit (*R* ^2^=0.33, *P<*0.08). Thus, there was a tendency to detect higher levels of response with greater numbers of vaccinated animals. However, the most interesting observation was that at levels of prevalence <10% (four out of 52 pigs over two Reps) it was possible to detect an increase in the 24 h mean Temp Diff following vaccination. The sensitivity of the Temp Diff parameter to prevalence was a function of using the maximum temperature as the response variable. Hypothetically, it only required one animal in a group to exhibit a higher than normal temperature for this to be detected by a thermal camera. This also explains why there was not a strong relationship between prevalence and the magnitude of the mean 24 h Temp Diff. Using the entire 24 h period after vaccination to define a response was relatively insensitive since the thermal response appeared to be mostly confined to a period of 3 to 20 h post-vaccination. Consequently, the 24 h mean response variable included temperature measurements that occurred outside of this time frame.

Provided that the barn temperature is kept within a narrow range, as was the case in the present study, there may be no need to correct the pig’s temperature measurement to take into account environmental effects. However, linear relationships and significant correlations between air temperature and radiated temperature of pigs have been demonstrated in the work of Geers *et al*. (1987) and Loughmiller *et al.* (2001). Consequently, under conditions in which there is a wide range in environmental temperature the effect on pig temperature would have to be taken into account.

There was a strong time of day effect on temperature that closely resembled the activity graph. Furthermore, clustering behaviour affected temperature measurements. Thus, it was important to account for these effects in modeling the response parameter. The Temp Diff parameter corrected for time of day effects because it paired temperature measurements made in real-time with time-matched measurements from prior control days. The clustering behaviour effect could be eliminated by only considering images in which the animals are arranged in a single cluster. However, this may not be the case under different environmental conditions since warmer environmental temperatures might reduce the prevalence of clustering behaviour. The image analysis software can be used to automatically count clusters. Given that both temperature and clustering behaviour were indicative of treatment effects it may be possible to combine these variables into a diagnostic index.

Since the ultimate goal is to utilize IRT technology to make real-time decisions in terms of disease detection and surveillance, a faster and more accurate assessment of temperature responses would be the running average in the Temp Diff variable, based on measurements of maximum pig temperatures. This response parameter has the potential to identify a thermal response within 6 to 10 h, while at the same time accounting for time-of-day effects such as clustering behaviour and circadian rhythms. These studies provide proof-of-concept that infrared images of groups of pigs can be used to detect febrile responses at low levels of prevalence, and thus have potential as a disease detection and surveillance tool in swine barns.
